# RAS protein activator like 2 promotes the proliferation and migration of pulmonary artery smooth muscle cell through AKT/mammalian target of Rapamycin complex 1 pathway in pulmonary hypertension

**DOI:** 10.1080/21655979.2021.1997879

**Published:** 2022-01-30

**Authors:** Sheng Hu, Youguang Zhao, Chenming Qiu, Ying Li

**Affiliations:** a Department of Pulmonary and Critical Care Medicine, The General Hospital of Western Theater Command, Chengdu, Sichuan, P.R. China; b Department of Urology, The General Hospital of Western Theater Command, Chengdu, Sichuan, P.R. China; c Department of Burn, The General Hospital of Western Theater Command, Chengdu, Sichuan, P.R. China; d Department of Geriatrics, The General Hospital of Western Theater Command, Chengdu, Sichuan, P.R. China

**Keywords:** Rasal2, pulmonary arterial hypertension, pulmonary artery smooth muscle cell, proliferation, migration

## Abstract

RAS protein activator like 2 (Rasal2) exerts pro-proliferative effect in several types of cells. However, whether Rasal2 is involved in the regulation of pulmonary artery smooth muscle cell (PASMC) remains unclear. In the current study, we explored the role of Rasal2 in proliferation and migration of PASMC during the development of pulmonary arterial hypertension (PAH). We found that the protein level of Rasal2 was increased in both pulmonary arteries of chronic hypoxia-induced pulmonary hypertension (CH-PH) mice and hypoxia-challenged PASMC. Overexpression of Rasal2 caused enhanced proliferation and migration of PASMC after hypoxia exposure. Mechanistically, we found elevated phosphorylation of AKT and two downstream effectors of mammalian target of Rapamycin complex 1 (mTORC1), S6 and 4E-Binding Protein 1 (4EBP1) after Rasal2 overexpression in hypoxia-challenged PASMC. Inactivation of mTORC1 abolished Rasal2-mediated enhancement of proliferation and migration of PASMC. Furthermore, we also demonstrated that AKT might act downstream of Rasal2 to enhance the activity of mTORC1. Once AKT was inactivated by MK-2206 application, overexpression of Rasal2 failed to further increase the phosphorylation level of S6 and 4EBP1. Finally, inhibition of AKT also blocked Rasal2-induced proliferation and migration in hypoxia-challenged PASMC. In conclusion, Rasal2 promotes the proliferation and migration of PASMC during the development of PAH via AKT/mTORC1 pathway.

## Introduction

Chronic pulmonary arterial hypertension (PAH) is a progressive pathophysiological syndrome characterized by abnormal pulmonary artery (PA) contraction, pulmonary vascular remodeling, accelerated vascular resistance, right ventricular failure and death [1,2]. PAH is associated with several pulmonary diseases, such as interstitial lung disease, chronic obstructive pulmonary disease and obstructive sleep apnea [3]. Among the above mentioned causes, hypoxia is being considered as one of the most crucial factors of PAH [4]. Many available drugs based on recovering the vasoconstrictive phenotype of PA fail to effectively improve the outcomes of PAH patients [5]. Therefore, searching novel therapeutic strategies for PAH is of great significance.

As the major component of vessel, pulmonary artery smooth muscle cell (PASMC) plays an important role in maintaining the normal function and structure of PA. There is a dynamic balance between proliferation and apoptosis in PASMC under physiological condition. However, long-term exposure to hypoxia causes excessive proliferation and migration of PASMC, leading to stenosis or occlusion of PA and further development of PAH [6]. The molecular mechanisms involved in the regulation of PASMC proliferation and migration are complex, including Mitogen Activated Protein Kinases and Adenosine 5‘-monophosphate (AMP)-activated protein kinase, etc [7,8]. Though classical drugs based on above-mentioned signaling pathways have been utilized, the morbidity and mortality of PAH are still at a high level. Therefore, searching novel molecular mechanisms in the regulation of PASMC proliferation and migration are particularly important.

Ras proteins play important roles in cellular proliferation and growth, including proliferation and migration of SMC [9,10]. Belonging to the RAS GTPase-activating protein family, RAS protein activator like 2 (Rasal2) is a critical factor in regulating RAS signaling pathway and is involved in many cellular processes [11]. Rasal2 is originally considered as a tumor-suppressor via its regulation of Wnt, Hippo and Ras signaling pathways [12]. However, recent researches indicate that the regulation of Rasal2 on cancer is multiple. Rasal2 can also exerts pro-tumor effect in several types of cancer [11,13]. A recent research revealed a novel role of Rasal2 in promoting adipogenesis and obesity-associated disorders [14]. Additionally, Rasal2 is also reported to negatively modulate angiogenesis in renal cell carcinoma [15], suggesting the potential role of Rasal2 in regulating vascular pathological and physiological functions. However, the role of Rasal2 in proliferation and migration of PASMC and PAH is unclear.

Here, we hypothesized that Rasal2 might be a novel molecular mechanism that is involved in PAH. In our current study, we observed elevated level of Rasal2 in both PA of chronic hypoxia-induced pulmonary hypertension (CH-PH) mice and PASMC after hypoxia stimulus. Upregulation of Rasal2 promoted hypoxia-induced proliferation and migration of PASMC. AKT/mammalian target of Rapamycin complex 1 (mTORC1) acted downstream of Rasal2. Inhibition of both AKT and mTORC1 abolished Rasal2-mediated phenotypic regulation of PASMC. Our study was expected to provide clues for finding new therapeutic targets of PAH.

## Materials and methods

### Animals

C57BL/6 J mice were purchased from Dashuo Animal Science and Technology (Chengdu, Sichuan, China). All animal procedures were conducted in accordance with the Institutional Animal Care and Use Committee and the Ethic Committee of The General Hospital of Western Theater Command (Chengdu, Sichuan, China). Mice were kept in a 12-hour light–dark cycle at 24°C and have free access to food and water. For PAH, mice were subjected to SU5416-hypoxia as previously described [16]. Briefly, 8–10-week-old male mice received subcutaneous injection of SU5416 (20 mg/kg; Sigma, St Louis, Missouri, USA) weekly and kept with chronic hypoxia (10% O_2_) in a ventilated chamber for 21 days. Normoxic control mice only received equivalent vehicle and were exposed to room air. Finally, mice were anesthetized with pentobarbital (100 mg/kg) and sacrificed for further experiments. Detection of right ventricular systolic pressure (RVSP) and right ventricular hypertrophy index (RVHI) were performed as previously described [17]. Briefly, mice were anesthetized with pentobarbital (30 mg/kg). A pressure transducer catheter (Millar Instruments, Houston, TX, USA) was then inserted into the right ventricle (RV) to measure the RVSP. The heart tissue of sacrificed mice were then isolated for calculating RVHI, which was determined by the mass ratio of RV/(left ventricle + septum).

### Cell culture and treatment

Primary cultured PASMC was obtained as previously described [18]. Briefly, PAs of mice were isolated and washed twice with ice-cold PBS. Then, the adventitia and connective tissue were carefully stripped off. The endothelium was gently removed via scratching the luminal surface with an elbow tweezer. The remaining media layer was cut into small pieces (1 mm^3^) and incubated with Dulbecco’s modified Eagle’s medium (DMEM; HyClone, Carlsbad, California, USA) supplemented with fetal calf serum (10%; Invitrogen, Carlsbad, California, USA), penicillin (100 units/ml) and streptomycin (100 mg/ml) in a humidified 5% CO2 incubator at 37°C. PASMC between passages 3 and 5 was used for further experiments. Normoxic PASMC was cultured under the condition of 21% O_2_, 5% CO_2_ and 74% N_2_ at 37°C for 6, 12 and 24 h. Hypoxic PASMC was cultured under the condition of 3% O_2_, 5% CO_2_ and 92% N_2_ at 37°C for 6, 12 and 24 h. RAPA (100 nmol/L) and MK2206 (1 μM) were utilized to treat cells for 6 h. The adenovirus expressing Rasal2 (Ad-Rasal2) and control viruses (Ad-con) were constructed according to the manufacturer’s instructions (Genechem; Shanghai, China). For transfer in PASMC, cell was transfected with adenovirus (3 pfu/cell) for 48–72 hours. For siRNA transfection, PASMC was incubated with the mixture of control siRNA or *Rasal2* siRNA (si-Rasal2) and 10 μL X-tremeGENE siRNA transfection reagent for 8 hours. Then, cells were cultured for another 48 hours before harvesting for RNA extraction. The sequence of siRasal2: CGGCGACTGGAG GAATATGAA [11].

### Western blotting

Extractions of PA and PASMC were lysed with RIPA buffer (Beyotime Institute of Biotechnology, Shanghai, China). The protein samples were then subjected to analysis of Western blotting as previous study described [19]. Primary antibodies against Rasal2 (cat 82481S, 1:1000), p-AKT^Thr308^ (cat 13038S, 1:1000), AKT (cat 9272S, 1:1000), p-S6^Ser235/236^ (cat 4858S, 1:1000), S6 (cat 14467S, 1:1000), p4EBP1^Thr37/46^ (cat 2855S, 1:1000), 4EBP1 (cat 9644 T, 1:1000) and β-actin (cat 4970S, 1:8000) were obtained from Cell Signaling Technology (Danvers, Massachusetts, USA).

### Immunofluorescence assay

Immunofluorescence of PASMC was conducted as previously described [19]. PASMC was seeded in a six-well plate and grown to 50%–60% confluence. After removal of cell culture media, cells were washed twice by PBS and fixed with 4% polyformaldehyde. PASMC was then incubated with 0.5% triton X-100 T for membrane breaking. After blocking, the cell was incubated with primary antibodies against SM α-actin and Ki-67 (1:1000; Cell Signaling Technology) overnight in dark at 4°C. PASMC was then rinsed by PBS and incubated with relative secondary antibodies (Alexa Fluor 594 F(ab’)-conjugated goat anti-mouse and Alexa Fluor 488-conjugated goat anti-rabbit (1:3000; Molecular Probes Inc., Eugene, Oregon, USA)) in dark for 1 h, respectively. The nuclei were stained with DAPI (5 mg/ml; VECTOR Labs, Burlingame, California, USA) for 5 s in the room temperature. Images were captured with immunofluorescent microscopy (Leica MPS 60, Germany).

### Transwell assay

Transwell assay of PASMC was performed as previously described [19]. 8‑μm pore Transwell chambers (Millipore, Darmstadt, Germany) were used for analysis of cell migration. PASMC was suspended with serum-free medium and seeded in the chambers at a density of 1 × 10^5^ cells per well. Serum-free DMEM was added into the lower wells of the chambers. After incubation under hypoxic conditions for 12 h at 37°C, the non‑migratory cells were wiped off from chamber membranes. Migratory PASMC was fixed with 4% paraformaldehyde for 15 min, rinsed twice with PBS and then stained with 1% crystal violet for 10 min. Finally, migratory cells were observed by a microscope (five fields per well were randomly selected).

### Statistical analysis

All data are expressed as mean ± standard deviation (SD). Comparisons between multiple groups were performed by analysis of variance (ANOVA) with an appropriate post hoc test. P < 0.05 was considered to be statistically significant.

## Results

In the current study, we investigated the effect of Rasal2 overexpression on the proliferation and migration of PASMC by utilizing the adenovirus carrying *Rasal2* (Ad-Rasal2). We confirmed that Rasal2 enhanced the proliferation and migration in hypoxia-challenged PASMC via AKT/mTORC1 pathway.

### The protein level of Rasal2 in CH-PH mouse model and hypoxia-challenged PASMC

**Figure 1.**
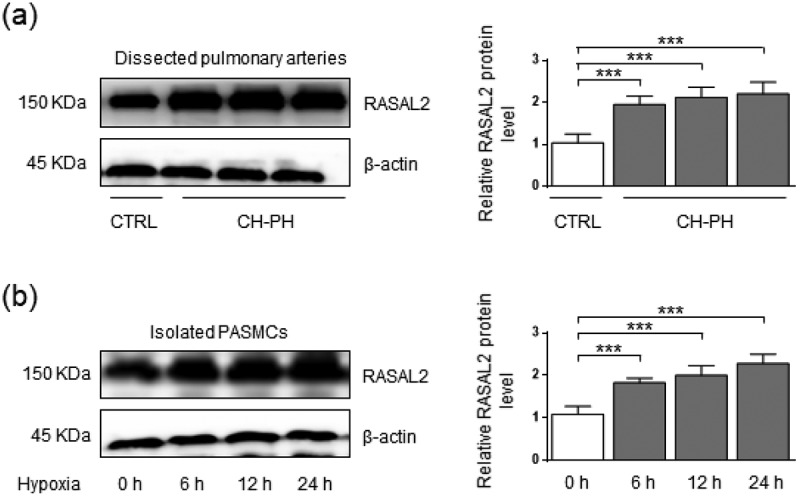
The protein level of Rasal2 is elevated in both dissected pulmonary arteries of pulmonary arterial hypertension (PAH) mice and hypoxia-challenged PASMC. (a) The protein expression levels of Rasal2 in dissected PAs of CH-PH mice were analyzed by immunoblotting (n = 5). (b) The protein expression level of Rasal2 in PASMC exposed to hypoxic conditions for different time courses was analyzed by immunoblotting (n = 5). Data are expressed as mean ± SD. *** indicates a significant difference of P < 0.001 between the two marked groups.

**Figure 2.**
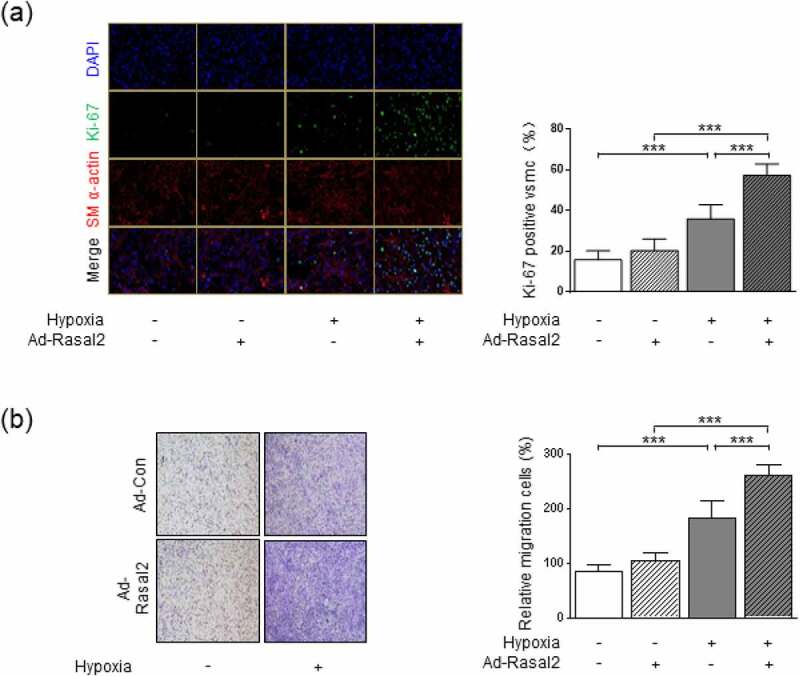
Rasal2 promotes hypoxia-induced proliferation and migration of PASMC. PASMC was transfected with the adenovirus expressing Rasal2 (Ad-Rasal2) or control viruses (Ad-Con) and cultured under normal/hypoxic conditions for 24 h. (a) Ki67 (green), SM α-actin (red) and DAPI (blue) staining was conducted by immunofluorescence (n = 5). Magnification 400 × . (b) Representative images showing the transwell assays for cell migration and relative quantification (n = 5). Magnification 100 × . Data are expressed as mean ± SD. *** indicates a significant difference of P < 0.001 between the two marked groups.

To determine whether Rasal2 is involved in the regulation of PAH, we first measured the relative protein level of Rasal2 in dissected PA of CH-PH mice. Compared to control mice, CH-PH mice showed significantly higher RVSP and RVHI (Supplementary Figure S1). As shown in Figure 1(a), increased protein expression level of Rasal2 was observed in CH-PH mice (Figure 1(a)). Since hypoxia-induced chronic phenotypic alteration of PASMC plays a crucial role in PAH, we also analyzed the expression of Rasal2 in PASMC after hypoxia stimulus. After 6, 12 and 24 hours of exposure to hypoxia, the protein expression of Rasal2 is both enhanced in PASMC (Figure 2(a)). These data suggest that upregulation of Rasal2 appears to be an important regulator for PAH development.

### Upregulation of Rasal2 promotes proliferation and migration in hypoxia-challenged PASMC

To further investigate the role of upregulation of Rasal2 in abnormal phenotypic transition of PASMC, we utilized Ad-Rasal2 to induce overexpression of Rasal2 in PASMC. As the result showed, transfection of Ad-Rasal2 led to significantly upregulation of Rasal2 in PASMC (Supplementary Figure S2). Phenotypic alteration of PASMC was evaluated by testing proliferation and migration. By using Ki-67 immunofluorescent staining, we observed a significant increase in number of Ki67-positive PASMC after 24 hours of hypoxia exposure (Figure 2(a)). Simultaneously, The increase in cell number induced by hypoxia was further enhanced by upregulation of Rasal2. PASMC migration was tested by utilizing transwell assay. Hypoxia led to enhanced migration of PASMC transfected with both Ad-Con (adenovirus carrying empty vector) and Ad-Rasal2 (Figure 2(b)). Additionally, overexpression of Rasal2 by Ad-Rasal2 transfection significantly accelerated the migration rate of PASMC under hypoxic condition. However, overexpression of Rasal2 did not cause significantly increase in number of migrated PASMC without hypoxia stimulus.

To further confirm whether hypoxia-induced upregulation of Rasal2 is responsible for abnormal proliferation and migration of PASMC, we also used siRNA strategy to test the effect of Rasal2. We found that silencing Rasal2 by siRNA significantly reduced the Ki-67-positive area and migratory capacity in hypoxia-challenged PASMC (Supplementary Figure S3). These data suggest that upregulation of Rasal2 may partly elucidate hypoxia-induced hyperproliferation and hypermigration in PASMC.

### mTORC1 is essential for Rasal2-mediated regulation on the phenotypic changes of PASMC

To elucidate the molecular mechanism by which Rasal2 accelerates the proliferation and migration of PASMC, the expression of classical molecules regulating growth of PASMC was validated after transfection of Ad-Rasal2. mTORC1 is a crucial complex for energy metabolism, which is involved in the development of PAH [20] and also in the phenotypic conversion of PASMC [21]. Ras is known to activate mTORC1 in several cell types [22]. However, whether Rasal2 modulates the activity of mTORC1 in PASMC remains unknown.

**Figure 3.**
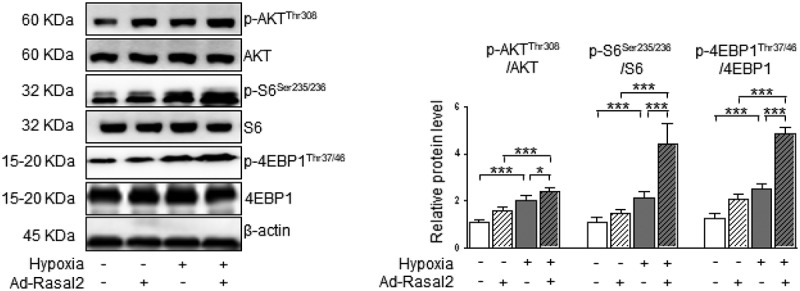
Rasal2 accelerates hypoxia-induced phosphorylation of S6 and 4EBP1 in PASMC. PASMC was transfected with Ad-Rasal2 or Ad-Con and cultured under normal/hypoxic conditions for 24 h. Images of immunoblotting and normalized expression levels of p-AKT^Thr308^, AKT, p-S6^Ser235/236^, S6, p-4EBP1^Thr37/46^, 4EBP1 and β-actin in PASMC are shown (n = 3). Data are expressed as mean ± SD. * and *** indicates a significant difference of P < 0.05 and P < 0.001 between the two marked groups, respectively.

**Figure 4.**
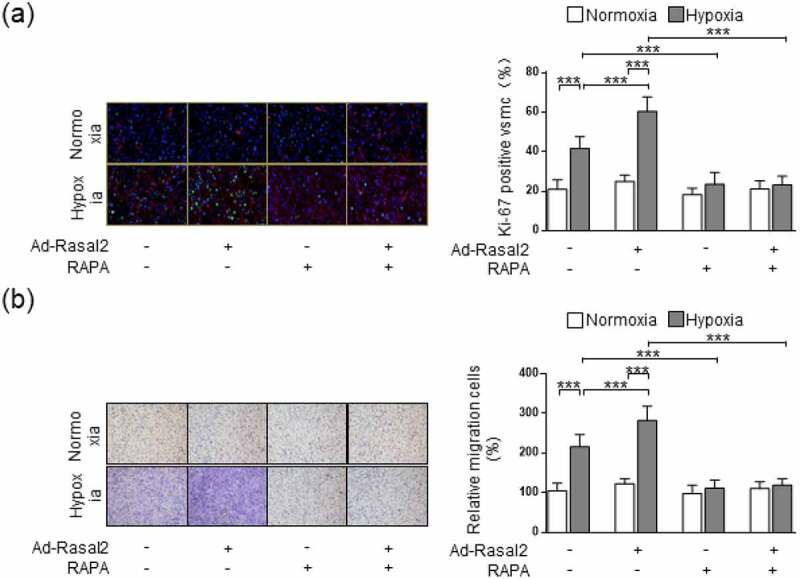
mTORC1 inactivation inhibits Ad-Rasal2-induced proliferation and migration in hypoxia-challenged PASMC. PASMC was firstly treated with RAPA (100 nmol/L) or equivalent vehicle for 6 h. Then, cells were transfected with Ad-Rasal2 or Ad-Con and cultured under normal/hypoxic conditions for another 24 h. (a) Ki67 (green), SM α-actin (red) and DAPI (blue) staining was conducted by immunofluorescence (n = 5). Magnification 400 × . (b) Representative images showing the transwell assays for cell migration and relative quantification (n = 5). Magnification 100 × . Data are expressed as mean ± SD. *** indicates a significant difference of P < 0.001 between the two marked groups.

As shown in our data, the phosphorylation of S6 and 4EBP1, the two downstream effectors of mTORC1, were both enhanced in hypoxia-challenged PASMC (Figure 3). Overexpression of Rasal2 caused a further increase in the phosphorylation of S6 and 4EBP1 after hypoxia stimulus. Additionally, silencing Rasal2 reversed hypoxia-induced phosphorylation of S6 and 4EBP1 in PASMC (Supplementary Figure S4). Next, we explored whether activation of mTORC1 is responsible for Rasal2-mediated regulation of PASMC under hypoxia condition. We utilized a classical mTORC1 inhibitor, rapamycin (RAPA), to rescue the abnormal mTORC1 activity induced by hypoxia. We found that increased Ki-67-positive cells in hypoxia condition was significantly reduced by RAPA (Figure 4(a)). More importantly, both hypoxia and overexpression of Rasal2 failed to further elevate the number of Ki-67-positive PASMC on the basis of RAPA application. The change of cell migration after inactivation of mTORC1 was also in accordance with that of cell proliferation. RAPA reduced the number of migrated PASMC challenged with hypoxia in both Ad-Con and Ad-Rasal2 group (Figure 4(b)). There was no significant difference in cell migration between hypoxia + RAPA + Ad-Con and hypoxia + RAPA + Ad-Rasal2 group. These data indicate that abnormal mTORC1 activation may act downstream of Rasal2 to achieve the modulation of PASMC phenotypic alteration.

### Phosphorylation of AKT mediates Rasal2-induced mTORC1 activation in PASMC

Since Rasal2 induced increased mTORC1 activity, proliferative and migratory capacity in PASMC, we next explored the upstream mechanism of mTORC1. As a sensor for cellular stress, mTORC1 is modulated by many protein kinases, which are sensitive to energy metabolism such as PI3K/AKT [23]. A previous research revealed that upregulation of Rasal2 was able to phosphorylate AKT to promote proliferation in hepatocellular carcinoma [24]. However, whether Rasal2 regulates mTORC1 activity via AKT in PASMC is still unrevealed.

**Figure 5.**
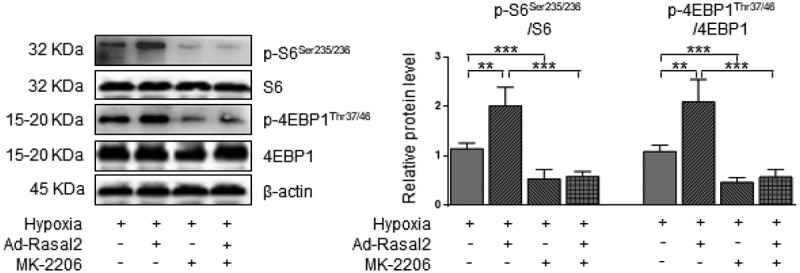
Rasal2-mediated phosphorylation of S6 and 4EBP1 in hypoxia-challenged PASMC was abolished by AKT inhibition. PASMC was firstly treated with MK2206 (1 μM) or equivalent vehicle for 6 h. Then, cells were transfected with Ad-Rasal2 or Ad-Con and cultured under normal/hypoxic conditions for another 24 h. Images of immunoblotting and normalized expression levels of p-S6^Ser235/236^, S6, p-4EBP1^Thr37/46^, 4EBP1 and β-actin in PASMC are shown (n = 4). Data are expressed as mean ± SD. ** and *** indicates a significant difference of P < 0.01 and P < 0.001 between the two marked groups, respectively.

In the current study, we found that hypoxia induced a significant increase in the AKT-Thr308 phosphorylation, which was further enhanced after overexpression of Rasal2 (Figure 3). Silencing Rasal2 inhibited hypoxia-induced phosphorylation of AKT in PASMC (Supplementary Figure S4). To further validate the relationship between AKT phosphorylation and mTORC1 activity, we utilized an AKT inhibitor, MK-2206 [25] to antagonize the phosphorylation of AKT induced by hypoxia or upregulation Rasal2. As shown in our data, both hypoxia and overexpression of Rasal2 failed to elevate the phosphorylation level of S6 and 4EBP1 after MK-2206 application (Figure 5). These data suggest that Rasal2-induced mTORC1 activation in PASMC is mediated by AKT phosphorylation.

### Blockage of AKT abolishes the induction of Rasal2 on phenotypic alteration of PASMC

**Figure 6.**
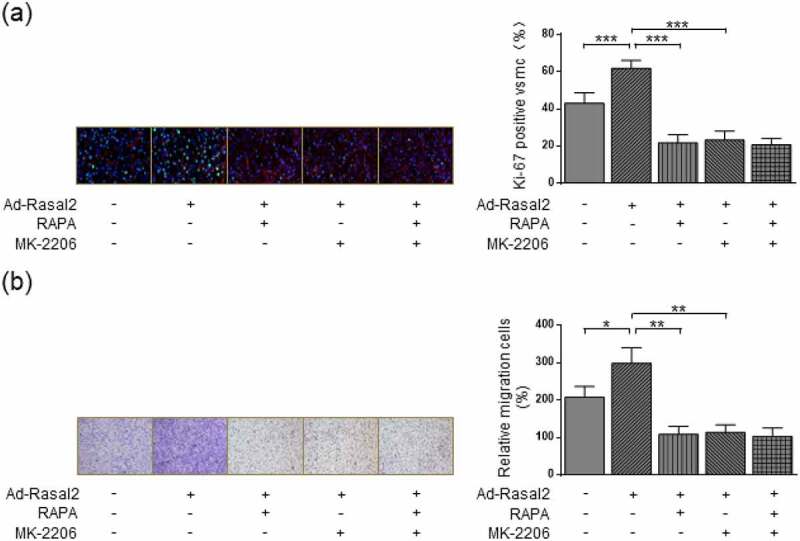
AKT inhibition suppresses Ad-Rasal2-induced proliferation and migration in hypoxia-challenged PASMC. PASMC was firstly treated with MK2206 (1 μM), RAPA (100 nmol/L) or equivalent vehicle for 6 h. Then, cells were transfected with Ad-Rasal2 or Ad-Con and cultured under normal/hypoxic conditions for another 24 h. (a) Ki67 (green), SM α-actin (red) and DAPI (blue) staining was conducted by immunofluorescence (n = 5). Magnification 400 × . (b) Representative images showing the transwell assays for cell migration and relative quantification (n = 5). Magnification 100 × . Data are expressed as mean ± SD. *, ** and *** indicates a significant difference of P < 0.05, P < 0.01 and P < 0.001 between the two marked groups, respectively.

Our research revealed that Rasal2 induced the mTORC1 activity of PASMC in a AKT-dependent manner. AKT is an important regulatory kinase during the occurrence and development of PAH [26]. Therefore, we further explored whether AKT inhibition impaired Rasal2 upregulation-induced proliferation and migration of PASMC. AKT inactivation by MK-2206 significantly attenuated the increase in Ki-67-positive PASMC induced by both hypoxia and Rasal2 upregulation (Figure 6(a)). MK-2206 showed similar inhibitory effect on PASMC proliferation to RAPA. Additionally, there was no significant difference in Ki-67-positive cells between hypoxia + Ad-Rasal2 + RAPA group and hypoxia + Ad-Rasal2 + RAPA + MK-2206 group. The enhanced capacity of cell migration induced by hypoxia or Rasal2 upregulation was also reduced after MK-2206 utilization (Figure 6(b)). After the blockage of mTORC1 activity by RAPA, MK-2206 failed to further lower the rate of PASMC migration. These data indicate that Rasal2-dependent AKT phosphorylation exerts a crucial role in promoting PASMC proliferation and migration via inducing mTORC1 activity.

## Discussion

Our current study sought to examine the role and mechanism of Rasal2 in the proliferation and migration of PASMC induced by hypoxia. First, the expression of Rasal2 was elevated in both PA of CH-PH mice and hypoxia-challenged PASMC. Second, overexpression of Rasal2 accelerated hypoxia-induced proliferation and migration of PASMC. Next, mTORC1 activation, characterized by increased phosphorylation of downstream kinases including S6 and 4EBP1 was associated with the enhanced proliferation and migration of PASMC after Ad-Rasal2 transfection. Recovery of mTORC1 activity by RAPA abolished the promotion of Rasal2 overexpression on hypoxia-induced phenotypic transition of PASMC. Finally, we demonstrated that the aforementioned effects of Rasal2 overexpression could be rescued by AKT inhibition. These results suggest that Rasal2 may be a crucial target for the prevention and treatment of PAH.

Belonging to the RAS GTPase-activating protein family, Rasal2 is a multifunctional factor that modulates a series of cellular processes, such as proliferation, lipid and glucose metabolism [12]. Rasal2 is also reported to be implicated in vascular regulation such as angiogenesis [15]. A previous study reveals that several differentially expressed kinases regulated by Rasal2, such as mitogen-activated protein kinases, are also crucial in PAH [27]. Our current study found that Rasal2 was also richly expressed within vessels. Nevertheless, the role of Rasal2 in PAH is rarely investigated. Characterized as excessive proliferation and migration, phenotypic transition of PASMC serves as a crucial process during the development of PAH. In the current study, Rasal2 in PASMC was significantly elevated after hypoxia exposure. External expression of Rasal2 further accelerated the hypoxia-induced proliferation and migration of PASMC. The pro-proliferation and pro-migration effects of Rasal2 on PASMC are also in accordance with its role in promoting the progress and metastasis of several tumor cells [11,13]. All these data suggest that Rasal2 promotes hypoxia‑induced PASMC proliferation and migration. However, the underlying molecular mechanism remains unclear.

mTORC1 serves as a crucial serine/threonine kinase in mediating various cellular processes, including proliferation, growth, survival and migration [28,29]. It has been demonstrated that mTORC1 is a core modulator during the development of PAH [30]. mTORC1 is also reported to induce the phenotypic transition of PASMC, which is the key pathological component of PAH. For example, chronic hypoxia induces phosphorylation of mTOR and S6, the downstream effector of mTORC1, to promote rat PASMC cell proliferation [31]. Additionally, mTOR signaling is also reported to be involved in SMC migration during neointima hyperplasia [32]. However, whether mTORC1 signaling is regulated by Rasal2 in PASMC is unknown. In our current study, we also observed the activation of mTORC1 in PASMC after hypoxia exposure. Overexpression of Rasal2 caused a further increase in mTORC1 activity. Therefore, we thought that mTORC1 might be an important target for the modulation of Rasal2 on PASMC proliferation and migration in PAH. We found that the enhanced capacity of proliferation and migration induced by Rasal2 overexpression in hypoxia-challenged PASMC was abolished by reducing mTORC1 activity. Our results suggest that mTORC1 is required in Rasal2-triggered phenotypic transition of PASMC.

As a sensor of cellular nutrient, redox and energy, mTORC1 is able to be activated by various mitogenic cytokines and growth factors [33,34]. The phosphoinositide 3-kinase (PI3K)/AKT is a critical signaling cascade that regulates cell proliferation [34]. Additionally, PI3K/AKT is also involved in the modulation of PASMC phenotypic transition and PAH development [35]. Previous research demonstrates that mTORC1 acts downstream of AKT to achieve its regulation of cellular processes [36]. We proposed that AKT might also mediate Rasal2-induced mTORC1 activation in PASMC. In this study, we demonstrated that overexpression of Rasal2 promoted the phosphorylation of AKT at site Thr308 in hypoxia-challenged PASMC. Furthermore, the enhanced phosphorylation of S6 and 4EBP1 was also attenuated after AKT inhibition. Meanwhile, AKT inhibition and mTORC1 inactivation showed similar suppressive effects on the proliferation and migration of PASMC after hypoxia exposure. AKT inhibition also abolished the promotive effect of Rasal2 on phenotypic transition of PASMC. Therefore, we revealed that Rasal2 induced PASMC proliferation and migration in an AKT/mTORC1-dependent manner.

## Conclusion

In summary, we provided evidence for aberrant expression of Rasal2 in CH-PH mice and revealed that Rasal2 induced proliferation and migration by activating AKT/mTORC1 pathway in hypoxic PASMC. Targeting Rasal2/AKT/mTORC1 might play a potential role in ameliorating the excessive proliferation and migration of PASMC in PAH patients.

## Supplementary Material

Supplemental Material

## Highlights

- Rasal2 is elevated in hypoxia-challenged PASMC and PA of CH-PH mice.
- Increased expression of Rasal2 promotes the proliferation and migration of PASMC.
- AKT/mTORC1 is essential for Rasal2-mediated regulation on PASMC.
