# Supplementary material for: RAS protein activator like 2 promotes the proliferation and migration of pulmonary artery smooth muscle cell through AKT/mammalian target of Rapamycin complex 1 pathway in pulmonary hypertension
Source: Bioengineered. 2022 Jan 30;13(2):3516–26. doi: 10.1080/21655979.2021.1997879 (PMC8973935; doi:10.1080/21655979.2021.1997879)
Supplement: Supplemental Material [file KBIE_A_1997879_SM4032.docx]

**RAS protein activator like 2 promotes the proliferation and migration of pulmonary artery smooth muscle cell through AKT/mammalian target of Rapamycin complex 1 pathway in pulmonary hypertension**

Sheng Hu^a,*^, Youguang Zhao^b,*^, Chenming Qiu^c^, Ying Li^d^

**Supplementary information**

**Supplementary Figure Legends**

Supplementary Figure S1. Chronic hypoxia induces significant increase in RVSP and RVHI. RVSP (a) and RVHI (b) of CH-PH mice were analyzed (n = 4). RVSP: right ventricular systolic pressure. RVHI: right ventricular hypertrophy index. Data are expressed as mean ± SD. ** and *** indicates a significant difference of P < 0.01 and P < 0.001 between the two marked groups, respectively.

Supplementary Figure S2. The protein level of Rasal2 is elevated in PASMC after Ad-Rasal2 transfection. The protein expression level of Rasal2 in PASMC after Ad-Rasal2 transfection was analyzed by immunoblotting (n = 4). Data are expressed as mean ± SD. *** indicates a significant difference of P < 0.001 between the two marked groups.

Supplementary Figure S3. Silencing Rasal2 inhibits hypoxia-induced proliferation and migration of PASMC. PASMC was transfected with siCon or siRasal2 and cultured under normal/hypoxic conditions for 24 h. (a) Ki67 (green) and DAPI (blue) staining was conducted by immunofluorescence (n = 4). Magnification 400×. (b) Representative images showing the transwell assays for cell migration and relative quantification (n = 4). Magnification 100×. Data are expressed as mean ± SD. ** and *** indicates a significant difference of P < 0.01 and P < 0.001 between the two marked groups, respectively.

Supplementary Figure S4. Silencing Rasal2 inhibits hypoxia-induced phosphorylation of S6 and 4EBP1 in PASMC. PASMC was transfected with siCon or siRasal2 and cultured under normal/hypoxic conditions for 24 h. Images of immunoblotting and normalized expression levels of p-AKT^Thr308^, AKT, p-S6^Ser235/236^, S6, p-4EBP1^Thr37/46^, 4EBP1 and β-actin in PASMC are shown (n = 3). Data are expressed as mean ± SD. ** and *** indicates a significant difference of P < 0.01 and P < 0.001 between the two marked groups, respectively.
